# The Effect of Hydrostatic Pressure on Enrichments of Hydrocarbon Degrading Microbes From the Gulf of Mexico Following the Deepwater Horizon Oil Spill

**DOI:** 10.3389/fmicb.2018.00808

**Published:** 2018-04-26

**Authors:** Angeliki Marietou, Roger Chastain, Felix Beulig, Alberto Scoma, Terry C. Hazen, Douglas H. Bartlett

**Affiliations:** ^1^Marine Biology Research Division, Center for Marine Biotechnology and Biomedicine, Scripps Institution of Oceanography, University of California, San Diego, La Jolla, CA, United States; ^2^Center for Geomicrobiology, Department of Bioscience, Aarhus University, Aarhus, Denmark; ^3^Department of Civil and Environmental Engineering, The University of Tennessee, Knoxville, Knoxville, TN, United States; ^4^Biosciences Division, Oak Ridge National Laboratory, Oak Ridge, TN, United States; ^5^Department of Microbiology, The University of Tennessee, Knoxville, Knoxville, TN, United States; ^6^Department of Earth and Planetary Sciences, The University of Tennessee, Knoxville, Knoxville, TN, United States; ^7^Institute for a Secure and Sustainable Environment, The University of Tennessee, Knoxville, Knoxville, TN, United States

**Keywords:** high pressure, Gulf of Mexico, Deepwater Horizon, oil spill, hydrocarbon-degrading microbes

## Abstract

The Deepwater Horizon oil spill was one of the largest and deepest oil spills recorded. The wellhead was located at approximately 1500 m below the sea where low temperature and high pressure are key environmental characteristics. Using cells collected 4 months following the Deepwater Horizon oil spill at the Gulf of Mexico, we set up Macondo crude oil enrichments at wellhead temperature and different pressures to determine the effect of increasing depth/pressure to the *in situ* microbial community and their ability to degrade oil. We observed oil degradation under all pressure conditions tested [0.1, 15, and 30 megapascals (MPa)], although oil degradation profiles, cell numbers, and hydrocarbon degradation gene abundances indicated greatest activity at atmospheric pressure. Under all incubations the growth of psychrophilic bacteria was promoted. Bacteria closely related to *Oleispira antarctica* RB-8 dominated the communities at all pressures. At 30 MPa we observed a shift toward *Photobacterium*, a genus that includes piezophiles. Alphaproteobacterial members of the *Sulfitobacter*, previously associated with oil-degradation, were also highly abundant at 0.1 MPa. Our results suggest that pressure acts synergistically with low temperature to slow microbial growth and thus oil degradation in deep-sea environments.

## Introduction

The 2010 Deepwater Horizon (DWH) accident in the Gulf of Mexico (GOM) is the largest and deepest marine spill recorded (The Federal Interagency Solutions Group, 2010). The well-head was located at 1544 m below sea surface (mbs); with a temperature of 4°C and a hydrostatic pressure of 15 megapascals (MPa). About one million metric tons of hydrocarbons were released into the Gulf (Joye et al., 2016). A microbial response to hydrocarbons was observed within weeks after the oil spill in both the oil plume and deep-sea sediments (Hazen et al., 2010; Kimes et al., 2013).

Rare members of the deep-sea *in situ* community responded rapidly to the oil spill (Kleindienst et al., 2016). It is estimated that the Gulf of Mexico basin hosts between 300 to 5000 natural oil seeps (Solomon et al., 2009) priming the autochthonous microbial community for oil degradation and might account for the quick response to the DWH oil spill.

Microbes from all three domains of life are able to biodegrade crude oil. There are at least 175 prokaryotic genera known to be able to degrade hydrocarbons under aerobic (i.e., water column) or anaerobic (i.e., sediments) conditions (Hazen et al., 2016). Interestingly, the microbial community response to the DWH oil spill changed over time with different genera dominating the microbial community in a step-wise manner. Initially, the plume was dominated by members of *Oceanospirillales* and *Pseudomonas*, which are able to consume different types of alkanes (Hazen et al., 2010). With a relative increase in aromatic hydrocarbons following partial capping, the microbial community in the plume shifted toward *Colwellia* and *Cycloclasticus*, which were potentially able to degrade (i) propane, ethane, butane, and (ii) BTEX (benzene, toluene, ethylbenzene, and xylene), respectively (Valentine et al., 2010; Dubinsky et al., 2013). Following the complete capping of the oil well the microbial community shifted again and was dominated mainly by methylotrophs, *Flavobacteria*, *Alteromonadaceae*, and *Rhodobacteraceae*, which were most likely involved in the degradation of the complex organic matter released from the dying bloom of the hydrocarbon degraders (Kessler et al., 2011). Members of the oil-degrading *Oceanospirillales*, *Colwellia*, and *Cycloclasticus* genera can be found in microbial communities of the cold and dark deep ocean around the world (Hazen et al., 2016).

The deep ocean is characterized by the absence of light, low temperatures and high pressure, all of which can have an important effect on microbial processes and therefore influence the rate and the extent of oil biodegradation at depth (Margesin and Schinner, 2001; Scoma et al., 2016c). The majority of the gas and certain components of the released oil were trapped at bottom waters (>1000 mbs) due to the physico-chemical effects of pressure, salinity and temperature at the well-head location (Redmond and Valentine, 2012). Initial reports of hydrocarbon degradation at deep sea pressure conditions (50 MPa) was reported on enrichments from sediments collected 4950 mbs in the Atlantic Ocean and incubated at 20°C (Schwarz et al., 1974). More recently, a piezotolerant alkane-degrading gamma-proteobacterium was isolated from 3475 mbs in the Mediterranean Sea was able to degrade alkanes with the same efficiency at low (0.1 MPa) and high (35 MPa) pressure at 20°C (Grossi et al., 2010). Several hydrocarbonoclastic isolates have been tested at various pressures while monitoring their ability to degrade oil constituents in order to understand the effect of pressure but at the same time overlooking the combined effect of low temperature as the studies were performed at room temperature (Schedler et al., 2014; Scoma et al., 2016a,b).

Recent expansion of deep-sea drilling necessitates a better understanding of the effect of low temperature and increasing pressure on microbial hydrocarbon degradation. Even though following the DWH oil spill an estimated 30% of the discharged hydrocarbons were degraded in deep water, most of the experimental approaches used to date have been performed at atmospheric pressure, excluding pressure as a parameter (Joye et al., 2016). The aim of this study was to explore the effect of pressure in shaping the autochthonous microbial community response to crude oil and identify potential hydrocarbon oxidizing microbes active at depths equal to or greater than 1500 m.

## Materials and Methods

### Sample Collection

Water samples were collected in the Gulf of Mexico on August 20, 2010 onboard R/V Ferrel at station BP-TN13-SS03 (27°27′4′′ N, 89°46′5′′ W, **Supplementary Figure S1**) with Niskin bottles attached to a CTD sampling rosette. The samples were collected 4 months after the spill and a month after the well was shut in. Water samples were collected at 1070 mbs (about 11 MPa) within an oxygen depletion zone [0.4 mg l^-1^ dissolved oxygen (DO)] suggestive of increased microbial activity. Cells were collected on 0.22-μm pore size membrane filters by filtering approximately 2.4 L of seawater. The filters were placed in 15 ml polyethylene bags and stored within a stainless steel pressure vessel at *in situ* pressure of 11 MPa at 4°C until further processing.

### Enrichments

The stored cells were resuspended from the filters by washing with 35 ml of APW medium (Coates et al., 1995). Macondo oil (MC-252, 1% v/v) from the DWH well [kindly provided by British Petroleum (BP)] was added to the cell suspension. The oil-amended cell suspensions were aliquoted into 5 ml polyethylene transfer pipette bulbs and incubated at *in situ* pressure (11 MPa) at 4°C. A month later 150 μl of the cultures was used to inoculate 15 ml of fresh APW medium amended with crude oil (1% v/v) and transferred to 15 ml polyethylene transfer pipette bulbs. Bulbs were incubated in independent replicates at atmospheric pressure (0.1 MPa), 15 MPa, and 30 MPa, along with un-inoculated negative controls (APW medium amended with crude oil, 1% v/v). Replicate bulbs were used to monitor cell number microscopically on days 0, 5, 15, 20, and 30. On day 30, 9 ml of the enrichments were filtered onto 0.2 μm Supor 25 mm membrane, placed in a sterile tube and stored at -80°C till further processing.

### Microscopy

Samples (1 ml) were fixed with paraformaldehyde (4% w/v), filtered onto 0.2 μm 25 mm black polycarbonate filters and stored at 4°C till further processing. The fixed cells were stained using 4′,6′-diamidino-2-phenylindole (DAPI) nucleic acid stain (Vector Laboratories, Inc., Burlingame, CA, United States) and viewed at 1000× magnification on an Olympus BX51 fluorescence microscope (Olympus).

### DNA Extraction and 16S rRNA Gene Analysis

The DNA extraction protocol used was optimized for obtaining DNA from small seawater volumes as described by Boström et al. (2004). The purified DNA was used as template for the amplification of bacterial 16S rRNA gene using the universal primers 27F (5′ AGA GTT TGA TYM TGG CTC AG 3′) and 1492R (5′ TAC GGY TAC CTT GTT ACG ACT 3′). The archaeal 16S rRNA gene was amplified *via* a two-step process: initially the purified DNA was used as template for a first round of amplification using primers 21F (5′ TCC GGT TGA TCC YGC CGG 3′) and 958R (5′ YCC GGC GTT GAM TCC AAT T 3′); the products of the first PCR were then used as a template for a second round of amplification using primers 21F and 517R (5′ ATT ACC GCG GCT GCT GG 3′). Following A-tailing of the PCR products, the pGEM-T Easy cloning vector system was used for the generation of 16S rRNA gene clone libraries. From each library 96 clones were submitted for direct sequencing (Beckman Coulter Genomics).

Quality control and trimming of the bacterial and archaeal 16S rRNA sequences was performed with Geneious Pro software (v4.8.5; Biomatters Limited, Auckland, New Zealand) using the option ScreenVector (UniVec High Sensitivity, Min BLAST alignment score 20), an error probability limit of 0.05, and a minimum post-trimming read-length of 400 bp. Further analysis of the quality-filtered sequences was performed using MOTHUR (v1.39.5; Schloss et al., 2009). Reads were screened for chimeras with the Uchime algorithm (Edgar et al., 2011), and aligned against the SILVA 123 database (Quast et al., 2013) for classification. For rarefaction analysis, sequences were grouped into OTUs with a distance cutoff of 0.03. Closely related sequences of cultured organism and environmental samples were identified and retrieved using NCBI’s Blast webserver^1^ with the blastn option (Altschul et al., 1990).

### Functional Gene Analysis

Target genes were detected using gene specific primers and relative abundance was measured using Power SYBR Master Mix (Life Technologies, Grand Island, NY, United States). Reactions were carried out in clear 384 plates using an ABI 7900 Real-Time PCR System (Life Technologies). The target genes were nahAc (Ac114F/Ac596R), alkB (alkB703F/alkB1573R), and xylE (xylEbf/xylEbr), while 16S rRNA gene (27F/1492R) was used as the internal standard (Salminen et al., 2008). The relative gene abundance was determined using the ΔΔ*C*t method (Livak and Schmittgen, 2001) and normalized to the 16S rRNA gene abundance, assuming one 16S rRNA gene copy and one functional gene copy per genome. The 15 MPa samples were considered as the reference for calculating relative gene expression changes.

### Gas Chromatography Mass Spectrometry (GC-MS)

The relative hydrocarbon composition of the enrichments at the end of the second round of incubation (day 30) was determined using GS-MS according to the method described by Hamamura et al. (2005) with the following modifications. Enrichment samples (5 ml) were extracted with dichloromethane (500 μl) by vigorous mixing at room temperature for 30 min. The mixture was allowed to sit for 2 h and then the solvent-oil phase (2 ml) was transferred to a new vial where another 500 μl of dichloromethane were added, shaken for 30 min and allowed to sit for 2 h. The solvent-oil phase was transferred to a clean vial where anhydrous sodium sulfate was added and allowed to stand for 30 min at room temperature to remove any residual water. Finally, the sample was collected and stored at -20°C till further processing. As control we used Macondo oil (1% v/v) in APW medium.

### Nucleotide Sequence Accession Numbers

The bacterial 16S rRNA sequences of the library clones were deposited in GenBank under accession numbers MG996148-MG996232 (0.1 MPa), MG996315-MG996398 (15 MPa), and MG996234-MG996314 (30 MPa). The archaeal 16S rRNA sequences were deposited in GenBank under accession numbers MH002460-MH002544 (0.1 MPa) and MG999967-MG999999, MH000001-MH000014 (15 MPa).

## Results and Discussion

**FIGURE 1:**
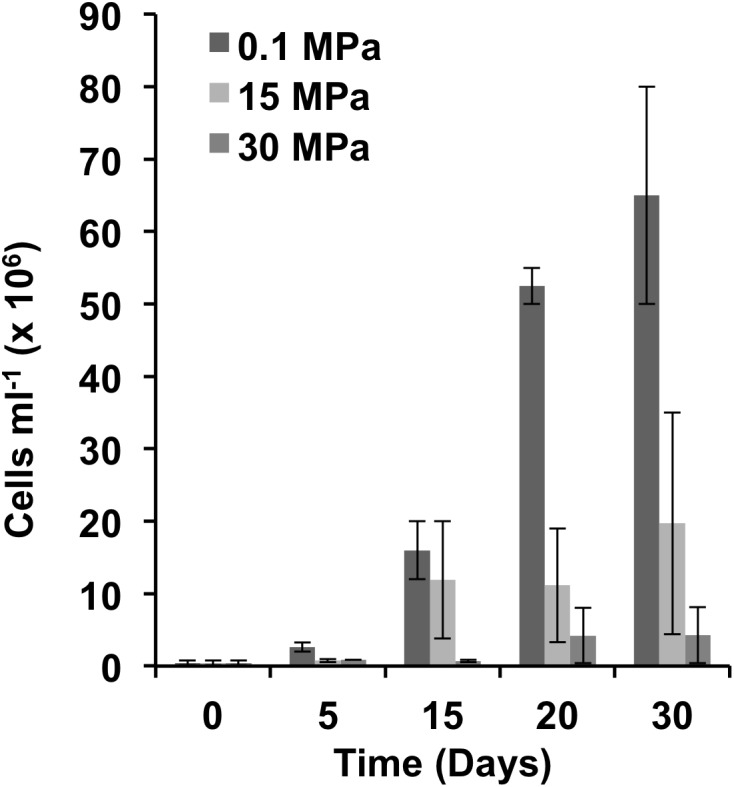
Cell abundance at different hydrostatic pressures. Cell counts were determined microscopically. Error bars indicate the standard deviation of the means (*n* = 2).

Following a pre-incubation at *in situ* pressure (11 MPa) and temperature (4°C) to increase cell biomass, a series of incubations were set up under three different pressure conditions replicating the pressure at (i) the sea-surface (0.1 MPa), (ii) the well head depth (15 MPa, 1500 mbs) and (iii) 3000 mbs (30 MPa). In samples incubated at atmospheric pressure growth started immediately with an average rate of 0.288 day^-1^ until day 20 when the rate dropped to 0.022 day^-1^ (**Figure 1**). At 15 and 30 MPa growth was observed following a delay of 5 and 15 days, respectively, at comparable growth rates to the atmospheric enrichments (**Figure 1**). Following 30 days of incubation we observed a 15-fold increase of cell numbers at the 0.1 MPa-enrichment and a 5-fold increase in the 15 MPa enrichment compared to the 30 MPa-enrichment (**Figure 1**). Maximum cell numbers were also observed at around 30 days for crude-oil enrichments prepared with GOM microbial communities at atmospheric pressure (Wang et al., 2016).

The lower cell numbers observed for the high pressure incubations (15 and 30 MPa) could suggest that the majority of the microbial community in the enrichments was composed of piezosensitive representatives. Incubation of the hydrocarbonoclastic, piezosensitive *Alcanivorax* sp. at 10 MPa (20°C) for 4 days resulted in significant reduction in the final growth yield (Scoma et al., 2016b). Other hydrocarbonoclastic piezotolerant isolates were able to grow at high pressure (35 MPa) at comparable rates and yields to those observed at 0.1 MPa (Grossi et al., 2010). However, both studies were not performed at *in situ* temperature. When high pressure incubations (50 MPa) were performed at temperatures reflecting the *in situ* conditions of the deep ocean (e.g., 4°C), growth rates and yields were significantly lower as compared to atmospheric pressure incubations (Schwarz et al., 1975). When incubated at 4°C, cultures enriched from sediments recovered from 4940 mbs and supplied with *n*-hexadecane reached a maximum growth within 28 days; however, when cells were incubated at 50 MPa growth was slower and maximum growth was achieved after 224 days, with yields 20-fold lower as compared to 0.1 MPa (Schwarz et al., 1975). Our results match the observations by Schwarz et al. (1975) suggesting that temperature has a significant impact on hydrocarbon degradation at depth. Low temperature alone is known to affect hydrocarbon degradation by (i) increasing oil viscosity, (ii) decreasing volatilization of short-chain alkanes, (iii) increase hydrocarbon water solubility, (iv) decreasing enzymatic activity (Leahy and Colwell, 1990). The synergistic effect of high pressure and low temperature appears to have an even more dramatic effect on the rates of microbial activity as extrapolated by our cell counts and the observations of Schwarz et al. (1975).

**FIGURE 2:**
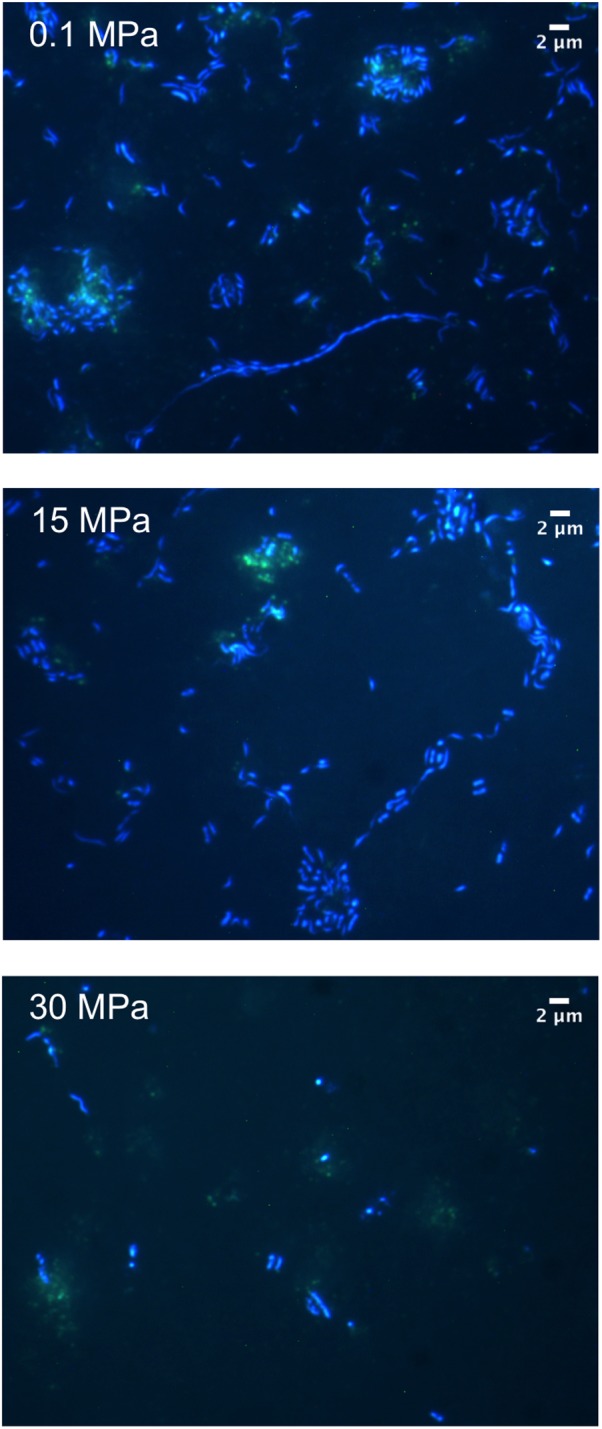
DAPI-stained micrographs of crude-oil enrichments at different hydrostatic pressures.

Microscopic examination of the enrichments revealed the presence of cells with a range of morphologies at 0.1 MPa including coccoid, bacilli, vibrio, spirillum, and filamentous (**Figure 2**). Cell length ranged from 0.33 μm for the smallest cocci to 1.36 μm for the majority of the rod-shaped cells, while some of the observed filaments reached 40 μm in length (**Figure 2**). No filaments were observed for samples enriched at 15 and 30 MPa (**Figure 2**). Using the auto-fluorescence properties of the oil we could observe increased cell clustering around oil flocks under all three pressure conditions (**Figure 2**). Microbial cell aggregation around oil droplets has been observed previously and is believed to be a type of nutrient conservation mechanism (Bælum et al., 2012). Cells appeared to aggregate immediately after the addition of oil and over time merged together to form flocs, which generally consist of extracellular polymeric substances (EPS), protein, oil, and bacteria (Bælum et al., 2012).

**FIGURE 3:**
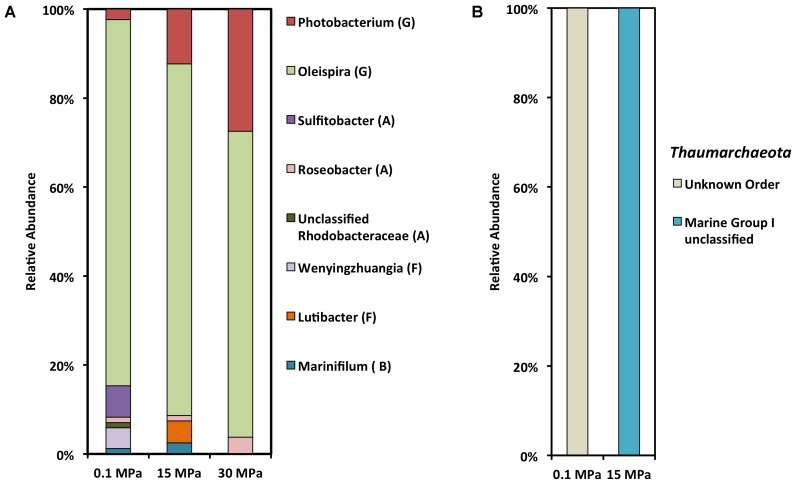
SSU rRNA gene clone library analysis of crude oil enrichments at different hydrostatic pressures. **(A)** Bacterial clone library (G, Gamma-proteobacteria; A, Alpha-proteobacteria; F, Flavobacteriia; B, Bacteroidia). **(B)** Archaeal clone library.

We used the 16S rRNA gene as a marker in order to determine the community composition of the enrichments (**Figure 3** and **Supplementary Figure S2**). Irrespective of the pressure condition, gamma-proteobacteria were the dominant phylotype, with alpha-proteobacteria being the second most abundant class at 0.1 MPa (**Figure 3A**). In previous studies there was a strong association with depth and the ratio between alpha- and gamma- proteobacteria, at depths greater than 100 mbs was <1 (King et al., 2013, 2015). Hazen et al. (2010) surveyed the autochthonous microbial response shortly after the DWH oil spill with a focus on the microbial diversity of a deep-sea plume extending from 1099 to 1219 mbs. Although Hazen et al. (2010) detected a higher number of cells in the deep-sea plume samples compared to the non-plume samples, the richness and diversity of the plume-associated community was low, with the majority of the OTUs restricted to a few γ-proteobacteria. Some of these formed a clade with known psychrophilic hydrocarbon degraders such as *Oleispira antarctica* (97% similarity, Hazen et al., 2010).

Under all three pressure conditions tested in this study *O. antarctica* RB-8 related OTUS dominated the gamma-proteobacterial community (**Figure 3** and **Table 1**). Within the gamma-proteobacteria we observed a decrease in *Oceanospirillales* and an increase in *Vibrionales* with increasing pressure. *Oceanospirillales* have been previously recovered from deep-sea oil plume enrichments at atmospheric pressure, while a piezophilic member of the *Oceanospirillales* capable of hydrocarbon degradation was previously isolated from the Puerto Rico Trench (Hazen et al., 2010; Cao et al., 2013). *O. antarctica* RB-8 is a psychrophilic, aerobic, aliphatic hydrocarbon degrader isolated from Antarctic seawater (Yakimov et al., 2003). At atmospheric pressure the most dominant alpha-proteobacterial OTUs belonged to *Sulfitobacter* related species (**Table 1**). *Sulfitobacter* sp. have been previously found at GOM beach sands impacted by the Deepwater Horizon oil spill (Kostka et al., 2011), while draft genome sequences of two *Sulfitobacter* sp. revealed the presence of genes on their genomes involved in aromatic hydrocarbon degradation (Mas-Lladó et al., 2014). Its growth in oil-contaminated seawaters at ambient pressure appears to be stimulated by low temperatures (Fasca et al., 2017). *Polaribacter* sp. and *Sulfitobacter* sp. related sequences were the most abundant among proteobacterial sequences at 22 MPa in a recent study examining the response of surface water microbial communities to low temperature and increasing pressure (Fasca et al., 2017).

**Table 1:** Phylogenetic affiliation of a representative set of 16S rRNA genes from the crude-oil enrichments at different hydrostatic pressures.

Sample	OTU	Pressure (MPa)	Closest relative	Identity (%)
Bacteria	01	0.1, 15, 30	*Oleispira antarctica* RB-8	99
	02	0.1, 15, 30	*Oleispira antarctica* RB-8	100
	03	0.1, 15, 30	*Photobacterium profundum* 3TCK	99
	04	0.1	*Sulfitobacter marinus* SW-265	99
	05	0.1	*Lutibacter oceani* 325-5	92
	06	30	*Halocynthiibacter arcticus* PAMC 20958	97
	07	15	*Lutibacter profundi* LP1	99
	08	0.1, 15	*Ancylomarina subtilis* FA102	99
	09	0.1, 15	*Loktanella maritima* KMM 9530	94
	10	15	*Lutibacter oceani* 325-5	97
	11	15	*Ancylomarina subtilis* FA102	97
	12	0.1	*Polaribacter huanghezhanensis* SM1202	95
Archaea	01	0.1	*Nitrosopumilus maritimus* SCM1	92
	02	15	*Nitrosopumilus maritimus* SCM1	90

Bacteriodetes OTUs within the *Marinifilacea* family were recovered from both the atmospheric and 15 MPa enrichments (**Figure 3**). *Marinifilum* sp. have been previously reported in water samples recovered from mesothermic oil fields, a shallow gas reservoir in the North Sea, and sediment samples following the DWH oil spill (Kryachko et al., 2012; Mau et al., 2015; Yang et al., 2016). Another Bacteriodetes OTU recovered only at 15 MPa was characterized as *Lutibacter* sp. (**Table 1**). *Lutibacter* sp. have been recovered previously from oil-polluted sediments in Patagonia for their ability to degrade polycyclic hydrocarbons (Isaac et al., 2013). Even though *Lutibacter* sp. OTUs were recovered at both 0.1 MPa and 15 MPa, interestingly the closest characterized match of the *Lutibacter* sp. OTU at 0.1 MPa was isolated from coastal marine sediments (Sundararaman and Lee, 2017), while the *Lutibacter* sp OTU recovered at 15 MPa was isolated from a deep-sea hydrothermal system (Le Moine Bauer et al., 2016). With increasing pressure we observed an increase in *Photobacterium profundum* related OTUs (**Table 1**). *Photobacterium* sp. are not usually recovered from oil-contaminated sites but members of the genus are well characterized piezophiles and have been previously recovered from various depths and pressure conditions. *Photobacterium* sp. have been previously found in naphthalene sediment enrichments from a harbor in Italy (Yakimov et al., 2005).

We were unable to detect any archaeal phylotypes at 30 MPa, while the archaeal OTUs detected at atmospheric and 15 MPa were in their majority unclassified Thaumarchaeota and those characterized belonged to Marine Group I (**Figure 3B**). Thaumarchaeota are abundant in diverse marine environments and play a role in the ocean nitrogen and carbon cycles (Techtman et al., 2017). Analysis of 16S rRNA diversity at several stations in the GOM a month prior to the oil spill revealed that Thaumarchaeota were among the more abundant OTUs in samples recovered from more than 100 mbs (King et al., 2013, 2015). The characterized archaeal OTUs had 90 to 92% identity with Candidatus *Nitrosopumilus* sp. which include autotrophic nitrifying marine archaea. The closest match for the most abundant recovered 16S sequence at 15 MPa was an uncultured clone recovered from the GOM at 400 m depth. Archaeal community members are negatively impacted by oil contamination as they do not appear to have an important role in oil degradation (Head et al., 2006). Even though hydrocarbon degrading halophilic and thermophilic Archaea have been reported as members of the microbial community following the DWH oil spill, their contribution to the bioremediation of the oil is considered negligible (Stetter et al., 1993; Tapilatu et al., 2010; Redmond and Valentine, 2012). Studies with *Nitrosopumilus maritimus* showed 20% inhibition of nitrite production at 1 ppb crude oil, while it was unable to utilize crude oil for growth (Urakawa et al., 2012). Similarly, Liu et al. (2017) reported a significant decrease in the archaeal population in oil-amended microcosm after only 72 h of incubation.

The majority of the laboratory-based studies following the DWH spill were performed at room temperature and atmospheric pressure. Redmond and Valentine (2012) set up crude oil enrichments at both room temperature and 4°C for 10 days and then analyzed the microbial community. Samples incubated at 4°C were predominated by *Colwellia* (87%), while samples incubated at room temperature were predominated by *Rhodobacteraceae* and *Colwellia* sp. constituted only 5% of the community. This further highlights the importance of temperature in shaping not only the potential degradation rates but also the composition of the microbial community in the DWH oil spill case (Redmond and Valentine, 2012). We were not able to detect any *Colwellia* sp. in our enrichments even though there are several characterized species with optimum growth pressures ranging from 60 to 120 MPa and optimal growth temperatures between 6 and 10°C (Yayanos et al., 1981; Deming et al., 1988; Nogi et al., 2004; Kusube et al., 2017). Genomic data and stable isotope probing experiments indicated that *Colwellia* was probably involved in the degradation of gaseous hydrocarbons (ethane, propane) and to a lesser extent to the degradation of aromatic hydrocarbons (Redmond and Valentine, 2012; Mason et al., 2014). Lab-scale experiments at 8°C and room temperature indicated *Colwellia* as a potential chemical-dispersant degrader (Kleindienst et al., 2015). However, all these possible carbon sources were low or absent in our experiments: even though volatile hydrocarbons constituted 24% of the released crude oil following the DWH spill (Reddy et al., 2012), our enrichments were prepared with the liquid oil fraction; the average half-life of aromatic hydrocarbons was more than 10 days while our enrichments were incubated for only 30 days quite possibly not allowing *Colwellia* to grow to sufficient numbers (Wang et al., 2016); the chemical dispersant Corexit 9500 used by Kleindienst et al. (2015) was absent in our tests.

**FIGURE 4:**
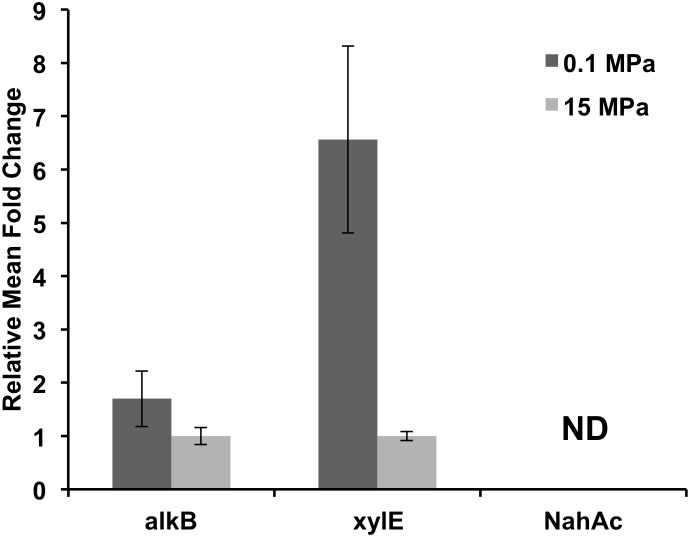
Relative functional gene abundance at 0.1 and 15 MPa. *alk*B, alkane hydroxylase; *xyl*E, catechol 2,3 dioxygenase; *nah*Ac, a naphthalene dioxygenase; ND, not detected.

We also examined the relative abundance of three functional genes in the 0.1 and 15 MPa enrichments (**Figure 4**), namely (i) *alkB*, an alkane hydroxylase, (ii) *xyl*E, a catechol 2,3 dioxygenase, and (iii) *nah*Ac, a naphthalene dioxygenase. The relative abundance of *alkB* genes did not change dramatically between the microbial communities at 0.1 and 15 MPa (**Figure 4**), in agreement with the consistently upregulated *alkB* expression reported in DWH plume samples vs. non-plume samples (Hazen et al., 2010; Lu et al., 2012; Mason et al., 2012; Rivers et al., 2013). We observed a sixfold increase in the relative *xylE* gene numbers in the 0.1 MPa enrichment as compared to the 15 MPa, while we were unable to detect any gene copies for *nah*Ac in either communities (**Figure 4**). When assuming that the relative gene abundance correlates well with the degradation potential of the community, it could be concluded that pressure did not affect significantly the alkane degrading capacity of the microbial community. However, there was a significant increase in the aromatic hydrocarbon degradation potential at atmospheric pressure compared to the *in situ* pressure conditions at 1500 m depth (**Figure 4**). It is unclear whether our inability to detect any *nah*Ac gene was due to the effect of pressure during the prolonged pre-incubation at 12 MPa or due to the short incubation period of the enrichments (30 days). In previous studies where hydrocarbon degradation was examined at low temperatures the half-life of alkane degradation was estimated from 2 to 10 days while for aromatic hydrocarbons it ranged from 2 to 37 days (Wang et al., 2016). Lower temperature (5°C) decreases significantly the rate by which aromatic hydrocarbons are degraded by introducing a lag phase of 15 days, while alkane degradation proceeds within a couple of days (Wang et al., 2016). The synergistic effect of high pressure and low temperature might have increased the lag phase of aromatic hydrocarbon degradation even further (Brakstad and Bonaunet, 2006). Moderate increase in pressure appears to have a stronger effect on aromatic hydrocarbon degradation compared to alkane. *Marinobacter hydrocarbonoclasticus* isolated from 3475 mbs was able to degrade *n*-hexadecane at both 0.1 and 35 MPa with no significant changes in growth or degradation rate (Grossi et al., 2010). The alkane degrader *Rhodococcus gingshengii* grew equally well on *n*-hexadecane at 0.1 and 15 MPa, while the aromatic hydrocarbon degrader *Sphingobium yanoikuyae* was unable to grow at pressures higher than 12 MPa (Schedler et al., 2014).

**FIGURE 5:**
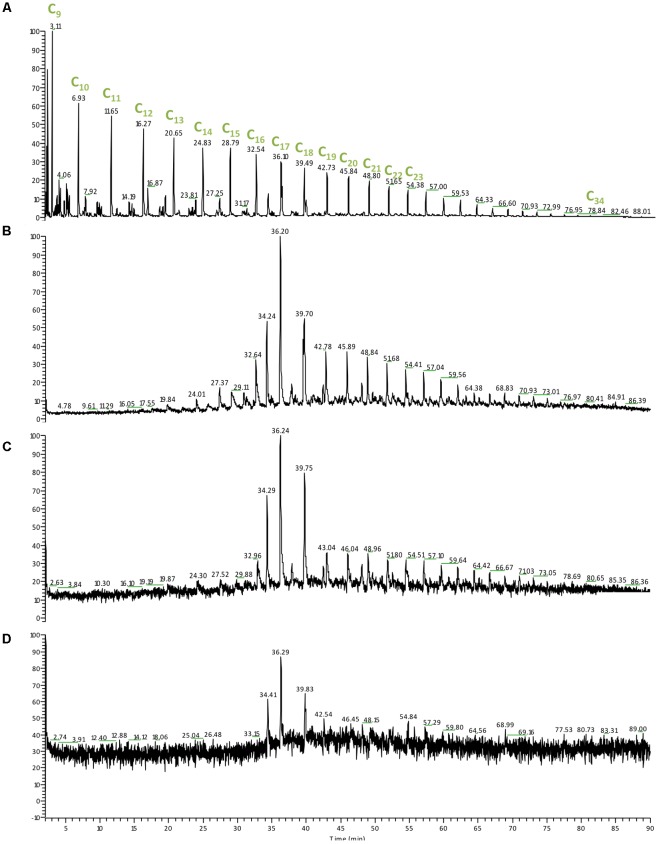
Gas chromatography mass spectrometry (GC-MS) chromatogram of the hydrocarbon relative abundance from the DWH enrichments at different pressures following 30 days of incubation at 4°C. **(A)** Crude oil control sample; **(B)** 0.1 MPa; **(C)** 15 MPa; **(D)** 30 MPa. *C*_x_, the number of carbons present in the hydrocarbon molecule detected.

The molecular mass and structure of the hydrocarbons affect the rate and extent of their biodegradation; as the molecular mass, ring number and alkyl-branching are increasing the degradation process becomes slower and further affected by environmental factors such as temperature and pressure (Bagby et al., 2017). We also monitored the changes of the oil composition in our enrichments (**Figure 5**). At 0.1 MPa there was complete loss of hydrocarbons lower than C_15_ as well as an increase in the relative abundance of C_17_ (**Figure 5**). Despite an increase at the baseline (un upward shift) at 15 MPa we observed complete loss of hydrocarbons lower than C_15_ as well as an increase in the relative abundance of both C_17_ and C_18_ (**Figure 5**). At 30 MPa we observed a significant shift at the baseline that masked the peaks lower than C_14_, however, complete loss of hydrocarbons lower than C_13_ could be detected (**Figure 5**). The hydrocarbon loss patterns observed under all three pressure conditions suggest that degradation of crude oil occurred under all three conditions and was mainly directed toward small molecular weight hydrocarbons (<C_13_). Similarly, in lab-simulations of the deep-sea plume was demonstrated that 6–13 carbon alkanes were degraded first with half-lives of 6–7 days (Hu et al., 2017). The preferential degradation of small molecular weight hydrocarbons could be associated with the reduced rates of degradation at higher pressures as observed previously by Schwarz et al. (1975), rendering the detection of high molecular weight and aromatic compounds degradation unlikely over the short course of incubation in this study. We observed that the microbial community response did not match the successional patterns reported previously following the DWH spill most likely due to slower degradation rates and a preferential degradation of small molecular weight hydrocarbons (<C_13_) shaping the microbial community analogously.

## Conclusion

The present study describes for the first time the microbial response to the DWH oil spill at *in situ* pressure and temperature conditions. Previous studies have shown that temperature has a significant effect on the final microbial community composition in response to oil (Redmond and Valentine, 2012). Our findings expand this observation to pressure and thus highlight which members of the microbial community are potentially able to degrade oil at increasing pressure. Despite observing the enrichment of an aliphatic hydrocarbon degrader (a relative of *O. antarctica* RB-8) and hydrocarbon degradation (GC-MS), growth yields as determined by optical density measurements were lower at high pressure (15 and 30 MPa). Furthermore, functional gene analysis suggests that the aromatic hydrocarbon degradation potential is reduced at high pressure (15 MPa).

Future microbial oil-degradation studies should take into account both pressure and temperature as important parameters when examining rates and activities at increasing depths since their synergistic effect has a significant influence on microbial rates and community dynamics. The observed lag in microbial activity and the lower growth yields observed at high pressures are suggestive of a significant slower microbial response at depth to anthropogenic oil input. More studies are required to understand the fate of spilled oil in the deep-sea. The present data suggest that oil entering deep waters will require significantly longer to be degraded by natural processes, resulting into longer persistence of the oil in the environment. Following the DWH oil accident, an estimated 30% of the spilled oil was degraded by the water-column microbial community (Joye et al., 2016), which begs the question of whether the response of the microbial community would have been equally efficient and fast at deeper waters? An estimated 4–31% of the DWH oil sunk to the seafloor at depths of 1300 to 1700 mbs (Valentine et al., 2014). The fate and impact of the sunken oil remains unclear to this day. Bioremediation by indigenous communities is currently the only (bio)technology available for oil degradation at deep sea, and the ultimate step in the clean-up of contaminated marine environments (Mapelli et al., 2017). Such indissoluble ties to microbial activity demand a deeper understanding of the actual cause-effect relationship of physicochemical stressors (e.g., temperature and pressure) on microbial hydrocarbon metabolism. As offshore deep-sea drilling is expected to increase in the near future (Maribus, 2014), and with limited maturity of deep-sea technologies claimed as a critical factor in the DWH accident (Jernelöv, 2010; Thibodeaux et al., 2011), this information is of importance to define up to date protocols to combat deep-sea oil spills.

## Author Contributions

AM and DHB designed the study. RC collected the samples and carried the initial sample handling. FB assisted with the 16S rRNA gene analysis. AM performed the experiments and prepared the manuscript under the supervision of AS, TCH, and DHB. All authors commented on the manuscript.

## Conflict of Interest Statement

The authors declare that the research was conducted in the absence of any commercial or financial relationships that could be construed as a potential conflict of interest.

## Footnotes

1 https://blast.ncbi.nlm.nih.gov/Blast.cgi

## Supplementary Material

FIGURE S1 Geographic location of sampling site (Google Maps). The red mark indicates the location of the sampling site, while the yellow mark indicates the location of the Macondo 252 (MC252) well.

FIGURE S2 Rarefraction curves of OTU diversity.
