# Corrigendum: The Effect of Hydrostatic Pressure on Enrichments of Hydrocarbon Degrading Microbes From the Gulf of Mexico Following the Deepwater Horizon Oil Spill

**DOI:** 10.3389/fmicb.2018.01050

**Published:** 2018-05-23

**Authors:** Angeliki Marietou, Roger Chastain, Felix Beulig, Alberto Scoma, Terry C. Hazen, Douglas H. Bartlett

**Affiliations:** ^1^Marine Biology Research Division, Center for Marine Biotechnology and Biomedicine, Scripps Institution of Oceanography, University of California, San Diego, La Jolla, CA, United States; ^2^Center for Geomicrobiology, Department of Bioscience, Aarhus University, Aarhus, Denmark; ^3^Department of Civil and Environmental Engineering, The University of Tennessee, Knoxville, Knoxville, TN, United States; ^4^Biosciences Division, Oak Ridge National Laboratory, Oak Ridge, TN, United States; ^5^Department of Microbiology, The University of Tennessee, Knoxville, Knoxville, TN, United States; ^6^Department of Earth and Planetary Sciences, The University of Tennessee, Knoxville, Knoxville, TN, United States; ^7^Institute for a Secure and Sustainable Environment, The University of Tennessee, Knoxville, Knoxville, TN, United States

**Keywords:** high pressure, Gulf of Mexico, Deepwater Horizon, oil spill, hydrocarbon-degrading microbes

In the original article, we neglected to acknowledge the Gulf of Mexico Research Initiative (231612-00), the Prince Albert II Foundation (Project 1265) and the National Science Foundation (1536776) for supporting DB.

Also, the name of one of the authors was incorrectly spelled in the reference for “Bauer, S. L. M., Roalkvam, I., Steen, I. H., and Dahle, H. (2016). *Lutibacter profundi* sp. nov., isolated from a deep-sea hydrothermal system on the Arctic Mid-Ocean Ridge and emended description of the genus Lutibacter. *Int. J. Syst. Evol. Microbiol*. 66, 2671–2677. doi: 10.1099/ijsem.0.001105” as “Bauer, S. L. M.”. It should be “Le Moine Bauer, S.”. The authors apologize for these errors and state that this does not change the scientific conclusions of the article in any way.

The original article has been updated.

## Conflict of interest statement

The authors declare that the research was conducted in the absence of any commercial or financial relationships that could be construed as a potential conflict of interest.

