# Supplementary material for: The Effect of Hydrostatic Pressure on Enrichments of Hydrocarbon Degrading Microbes From the Gulf of Mexico Following the Deepwater Horizon Oil Spill
Source: Front Microbiol. 2018 Apr 26;9:808. doi: 10.3389/fmicb.2018.00808 (PMC5932198; doi:10.3389/fmicb.2018.00808)
Supplement: FIGURE S1 — Geographic location of sampling site (Google Maps). The red mark indicates the location of the sampling site, while the yellow mark indicates the location of the Macondo 252 (MC252) well. [file Image_1.PDF]

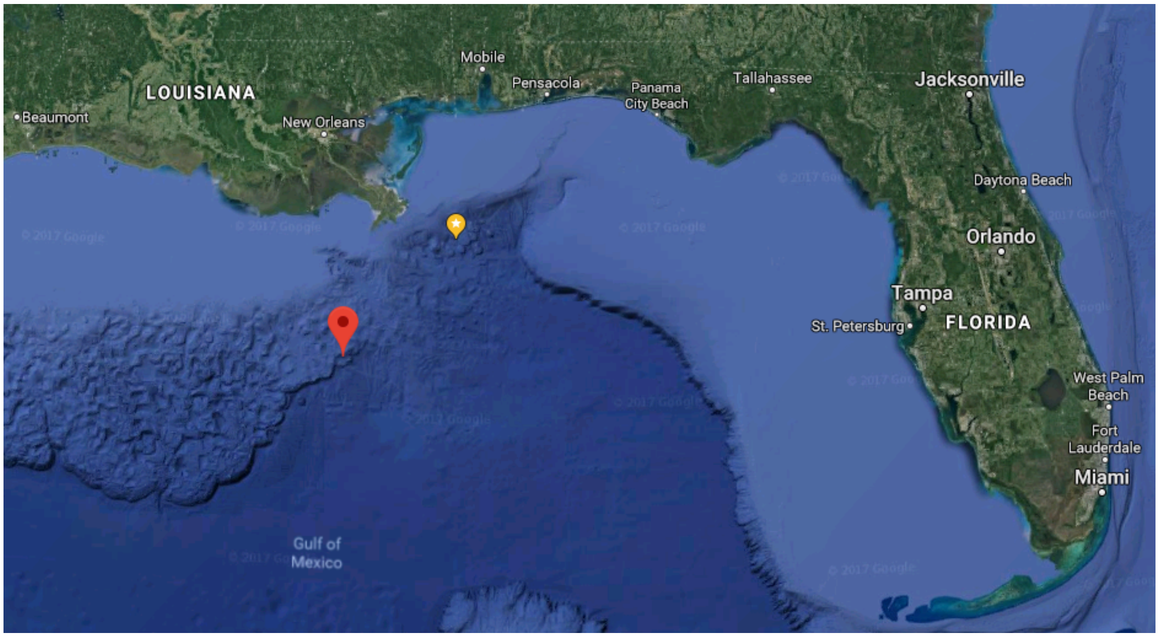

Supplementary Figure 1. Geographic location of sampling site (Google Maps). The red mark indicates the location of the sampling site, while the yellow mark indicates the location of the Macondo 252 (MC252) well.
