# Supplementary figures and images for: The Effect of Hydrostatic Pressure on Enrichments of Hydrocarbon Degrading Microbes From the Gulf of Mexico Following the Deepwater Horizon Oil Spill
Source: Front Microbiol. 2018 Apr 26;9:808. doi: 10.3389/fmicb.2018.00808 (PMC5932198; doi:10.3389/fmicb.2018.00808)

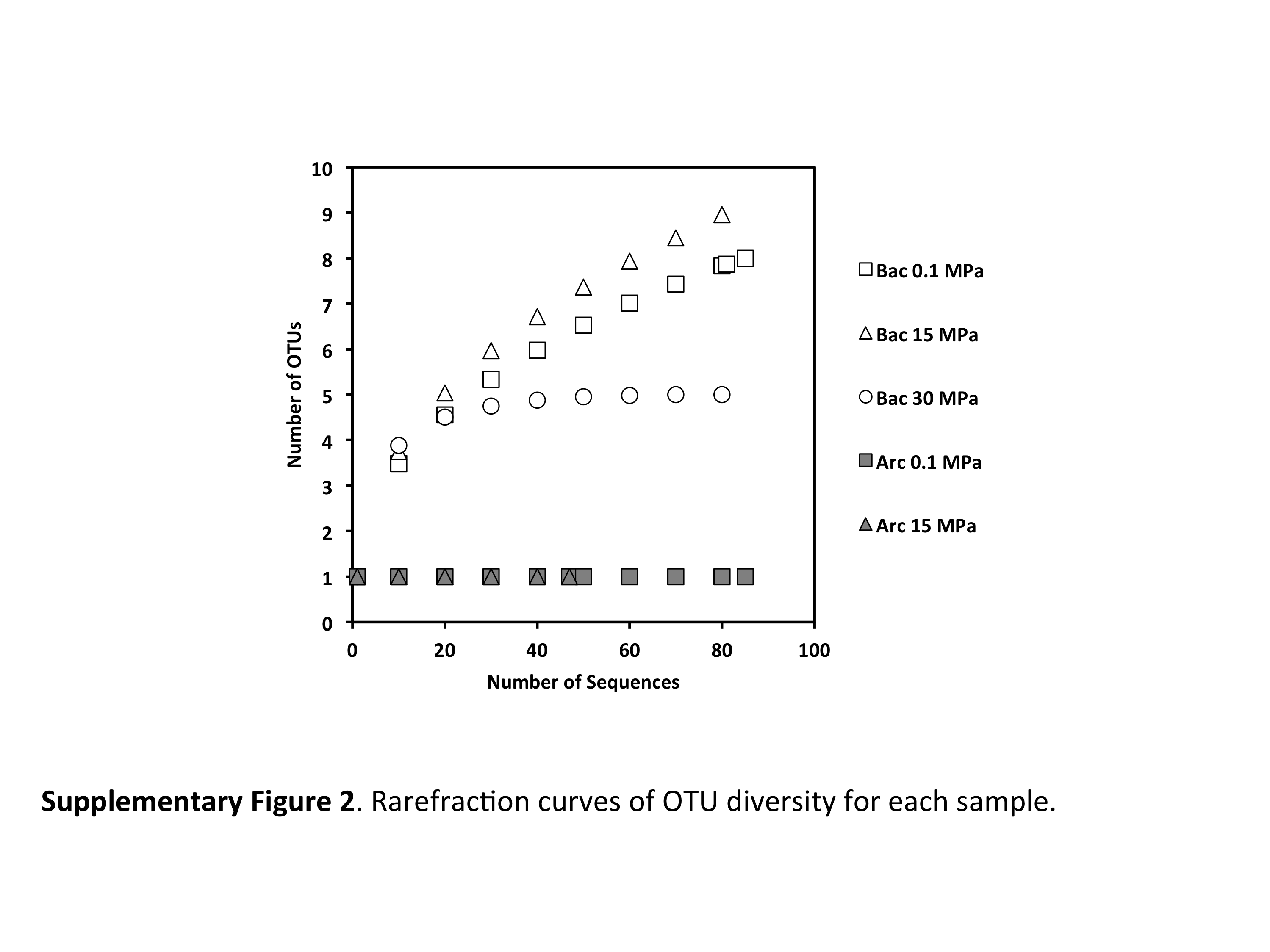

Supplement: FIGURE S2 — Rarefraction curves of OTU diversity. [file Image_2.TIF]
